# Finding Significant Hits in Networks: a network-based tool for analyzing gene-level *P*-values to identify significant genes missed by standard methods

**DOI:** 10.1093/bib/bbag061

**Published:** 2026-03-08

**Authors:** Sandeep Acharya, Vaha Akbary Moghaddam, Wooseok J Jung, Yu S Kang, Shu Liao, Michael A Province, Michael R Brent

**Affiliations:** Division of Computational and Data Sciences, Washington University, 1 Brookings Dr, St. Louis, MO 63130, United States; Division of Statistical Genomics, Washington University School of Medicine, 4515 McKinley Ave, St. Louis, MO 63110, United States; Department of Computer Science and Engineering, Washington University, 1 Brookings Dr, St. Louis, MO 63130, United States; Department of Computer Science and Engineering, Washington University, 1 Brookings Dr, St. Louis, MO 63130, United States; Department of Computer Science and Engineering, Washington University, 1 Brookings Dr, St. Louis, MO 63130, United States; Division of Statistical Genomics, Washington University School of Medicine, 4515 McKinley Ave, St. Louis, MO 63110, United States; Department of Computer Science and Engineering, Washington University, 1 Brookings Dr, St. Louis, MO 63130, United States

**Keywords:** gene prioritization, relaxed significance thresholds, network-based analysis, novel gene discovery, replicable gene-trait associations

## Abstract

Finding Significant Hits in Networks (FISHNET) uses prior biological knowledge, represented as gene interaction networks and gene function annotations, to identify genes that do not meet the genome-wide significance threshold but replicate, nonetheless. Its input is gene-level *P*-values from any source, including omicsWAS, aggregation of genome-wide association studies *P*-values, CRISPR screens, or differential expression analysis. It is based on the idea that genes whose *P*-values are low purely by chance are distributed randomly across networks and functions, so genes with suggestive *P*-values that cluster in densely connected subnetworks and share common functions are less likely to reflect chance and more likely to replicate. FISHNET combines network and function analysis with permutation-based *P*-value thresholds to identify a small set of exceptional genes that we call FISHNET genes. Applied to 11 cardiovascular risk traits, FISHNET identified 19 gene-trait relationships that missed genome-wide significance thresholds but, nonetheless, replicated in an independent cohort. The replication rate of FISHNET genes matched that of genes with lower *P*-values. FISHNET identified a novel association between *RUNX1* expression and HDL that is supported by experimental evidence that *RUNX1* promotes white fat browning, which increases HDL cholesterol levels. FISHNET also identified an association between *LTB* expression and BMI that is supported by experimental evidence that higher LTB expression increases BMI via activation of the LTβR pathway. Both associations failed genome-wide significance thresholds, highlighting FISHNET’s ability to uncover meaningful relationships missed by traditional methods. FISHNET software is freely available at https://brentlab.github.io/fishnet/.

## Introduction

The primary goal of hypothesis testing is to determine whether an observation made in a sample from a population is likely to be true in the entire population. Statistical hypothesis testing is typically used to minimize the risk of false positive findings at the cost of substantial risk of false negatives. The false-negative risk is magnified by multiple testing correction (typically Bonferroni [1, 2] or Benjamini-Hochberg [3, 4]), particularly when many tests are carried out, as is the case in genome-wide association studies (GWAS) [5, 6], transcriptome-wide association study (TWAS) [7, 8], and other omics-WAS [9, 10]. Moreover, when effect sizes are small, as is typical in genetics, very large samples are needed to reach statistical significance. Thus, there is an urgent need for methods that can identify true, population-wide omics-trait relationships that do not meet Bonferroni or Benjamini-Hochberg significance thresholds. Such methods can be evaluated empirically by replication studies, in which additional samples are drawn from the population.

We propose Finding Significant Hits in Networks (FISHNET), a new method for finding exceptional gene-trait associations that replicate at a higher rate than other associations with the same *P*-values. FISHNET integrates results from gene-level summary statistics with prior biological knowledge represented as networks. It can use gene-level summary statistics from GWAS, TWAS with measured or predicted gene expression levels, proteome-wide association studies, RNA-Seq experiments, functional genetics screens, or any other source. It uses gene–gene interactions from co-expression networks, protein–protein interaction (PPI) networks, or other networks, together with gene function annotations from Gene Ontology (GO) [11]. We hypothesize that genes whose *P*-values are low by chance are distributed randomly across biological networks and functions. Thus, when genes with low *P*-values cluster in densely interconnected subnetworks (network modules) and share common functions, they are less likely to reflect sampling error and therefore more likely to replicate in new samples. FISHNET combines network module enrichment analysis [12] and GO over-representation analysis [13] with permutation-based significance thresholds to identify a small set of exceptional, trait-influencing genes that we call FISHNET genes.

FISHNET is specifically designed to identify gene-trait associations that replicate in independent samples at a higher rate than other associations with similar *P*-values. In addition to prioritizing genes that meet traditional significance thresholds, it identifies genes below these thresholds that replicate at a similar rate, enabling thresholds to be relaxed. Notable features include its permutation-based significance testing, its automated testing of model assumptions, and its use of GO biological process annotations in addition to networks. These annotations, which are manually curated and reflect a large body of literature [11], are complementary to networks, which are typically generated from high-throughput data. Another reason for supplementing networks with GO analysis is that network modules are identified computationally [14], based on network connectivity, so there is no guarantee that two genes in the same module share a common function.

This paper makes four contributions. First, it introduces the FISHNET algorithm and software. The software is freely available, easy to install, and easy to deploy across a wide range of computing environments. Second, it evaluates the replicability and reproducibility of FISHNET genes using 43 sets of GWAS gene-level summary statistics [14]. Evaluation was carried out on nine combinations of networks and algorithms for finding modules, identifying the most useful combinations. Third, using the best networks and the best module detection algorithm, it identifies FISHNET genes across 11 traits associated with cardiovascular risks in the Long Life Family Study (LLFS) cohort and performs the replication analysis in the Framingham Heart Study (FHS) cohort (See acknowledgements) [15, 16]. The results show that FISHNET genes have a better replication rate than non-FISHNET genes with similar *P*-values. Fourth, this paper introduces metrics to assess whether user inputs—network modules and gene-level summary statistics—violate the model assumptions.

## Methods

### Gene-level summary of genome-wide association studies

Gene-level summary statistics were obtained from 185 meta-analyses of GWAS collected for the 2019 Disease Module Identification DREAM Challenge [14]. The SNP-level summary statistics were aggregated to gene-level statistics using PASCAL [12]. PASCAL uses the sum of chi-squared approach to calculate a gene-level *P*-value. To create discovery and replication set pairs for replication analysis, we used only traits that had more than one study with completely or partially independent cohorts. Additionally, we removed studies where the genotyped SNPs did not cover all chromosomes in the genome. After these exclusions, we retained gene-level summary statistics from 43 GWAS. There were 17 traits with exactly two GWAS datasets—Bipolar disorder, BMI, BMI (male), BMI (female), Coronary artery disease, Fasting glucose, Height, Hip circumference (male), Hip circumference (female), Molecular degeneration, Rheumatoid arthritis, Total cholesterol, Waist circumference (male), Waist circumference (female), Waist-hip ratio (male), Waist-hip ratio (female), and Schizophrenia. For High-density lipoprotein (HDL), Low-density lipoprotein (LDL), and Triglycerides, there were three GWAS studies, two of which we used as discovery sets, with the third as the replication set for both. The final dataset consisted of 23 discovery and replication set pairs, each with gene-level GWAS summary statistics. The characteristics of each dataset can be found in Supplementary Table S1.

### Long Life Family Study

The LLFS is a longitudinal family study that enrolled families enriched for exceptional longevity to discover genetic factors contributing to healthy aging. LLFS enrolled 4953 participants in 539 pedigrees, primarily of European ancestry (99%). The recruitment procedure and enrollment criteria of the LLFS participants have been previously described [17, 18]. The data generated in the study includes gene expression levels from blood and biomarkers of health and aging. We focused on 11 traits associated with cardiovascular risks spanning four categories: pulmonary (forced expiratory volume, forced vital capacity, and the ratio of the two), lipids (HDL, LDL, triglycerides, total cholesterol), anthropometric (BMI, BMI-adjusted waist), and cardiovascular (pulse, ankle-brachial index) [19–23].

### Long Life Family Study RNA-seq and transcriptome wide association

RNA extraction and sequencing were carried out by the McDonnell Genome Institute at Washington University. Total RNA was extracted from PAXgene™ Blood RNA tubes using the Qiagen PreAnalytiX PAXgene Blood miRNA Kit (Qiagen, Valencia, CA). The RNA-Seq data were processed with the nf-core/rnaseq pipeline version 3.3 using STAR/RSEM and otherwise default settings (https://zenodo.org/records/5146005). The RNA-Seq data QC steps and the gene expression level adjustment model used in this study have been previously described by Acharya *et al*. [8] who performed TWAS on the same 11 traits using the data from the first clinical exam in LLFS. Since our previous publication, the dataset has grown. Depending on the trait, the number of participants with both RNA-Seq and trait data now ranges from 879 to 1667. The adjustment steps for all 11 traits are described in [8]. Supplementary Table S2 shows the characteristics of study participants for covariates and 11 cardiovascular risk traits.

For each trait, the adjusted gene expression residuals were used as a predictor and the adjusted trait residuals were used as a response variable in a linear mixed model implemented in MMAP [24]. A kinship matrix generated by MMAP from the LLFS pedigree was used to account for family relatedness. For traits with genomic inflation factor > 1.1, the *P*-values were adjusted by using BACON [25]. The same RNA-Seq processing and trait adjustment steps were applied for replication in the FHS dataset, where the number of participants with data for each trait ranged from *n =* 1080 to *n =* 1380.

### Module enrichment analysis and Gene Ontology over-representation analysis

**Figure 1:**
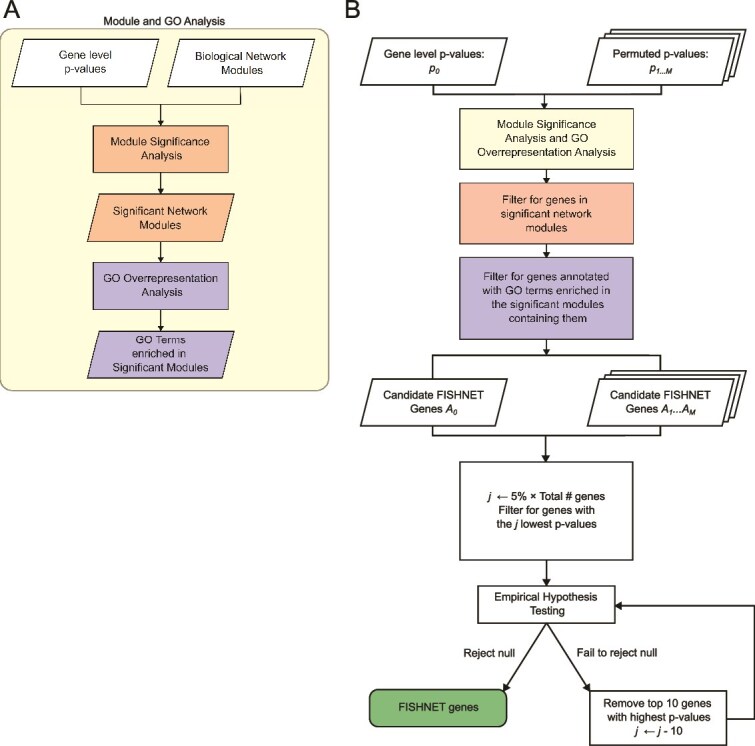
(A) The first step in the FISHNET workflow. The gene-level *P*-values are input into module significance analysis. Module significance analysis outputs significant modules and their *P*-values. Gene ontology over-representation analysis identifies biological processes with significant over-representation among genes in each significant module. (B) The workflow used to identify FISHNET genes. For details of the permutation-based hypothesis testing, see Methods.

First, in each selected gene–gene interaction network, network modules (highly connected subnetworks) were identified by three algorithms from the Disease Module Identification DREAM Challenge [14], designated as R2 (based on random walk), K1 (based on kernel clustering), and M2 (based on modularity optimization). The MONET software incorporates these three modularization algorithms. Each algorithm takes in a gene network and returns modules of highly interconnected genes. The source code and user documentation are available at https://github.com/BergmannLab/MONET. The modules identified by MONET in this work were from STRING functional PPI network [26], InWeb physical PPI network [27], and a gene co-expression network from Gene Expression Omnibus [28, 29]. In addition to aggregating SNP-level *P*-values to gene-level, the PASCAL software package provides separate functionality to aggregate gene level *P*-values to module level [12]. As such, after identifying network modules, GWAS or TWAS gene-level summary statistics and sets of genes in network modules were fed into PASCAL’s module enrichment algorithm [12]. Figure 1A depicts the inputs and outputs of module enrichment analysis and GO over-representation analysis. After modules were identified, the connections between genes in modules were not used. PASCAL’s module enrichment algorithm outputted sets of genes in modules significantly enriched for genes with low *P*-values, adjusted for the total number of modules tested using Bonferroni correction. GO over-representation analysis was done on the set of genes in each enriched module by using WebGestaltR (version: 0.4.6) with the following configuration: (organism: hsapiens, method: ORA, enrichDatabase: GO Biological Process, FDRMethod: BH, FDRThreshold = 0.05) [13]. The affinity propagation feature in WebGestaltR was used to eliminate GO biological processes with highly overlapping member genes, thereby reducing the multiple testing burden and improving computational performance.

### Finding Significant Hits in Networks algorithm

Figure 1B depicts the FISHNET algorithm. FISHNET is run separately for each combination of traits, networks, and module detection algorithms. Genes are considered caught in the FISHNET if they:

1. Are in a network module unusually enriched for genes with low *P*-values (PASCAL’s module enrichment analysis). This passes only genes that work together with other trait-implicated genes. And,
2. Are annotated by a GO biological process term that is enriched in their module (GO over-representation analysis). This passes only genes that work together as a part of a common biological process. And,
3. Are among the top *j* genes ranked by *P*-values. *j* starts at 5% of genes. If all genes’ *P*-values are under the null model, they should be uniformly distributed, in which case the top 5% corresponds to a nominal *P*-value threshold of 0.05; if the *P*-value distribution is inflated, the top 5% will correspond to a *P*-value threshold lower than 0.05.
  a If using the optional permutation-based hypothesis testing described below, *j* is reduced until specified significance criteria are met.

### Permutation-based null model: empirical hypothesis testing

Null hypothesis: The number of candidate FISHNET genes is not substantially higher than expected at random.

PASCAL’s module enrichment analysis pipeline monotonically transforms the genes’ *P*-value distribution into a chi-squared distribution. This process relies only on the ranks (quantiles) of the genes when ranked by their *P*-values. Therefore, FISHNET works as follows (also shown in Fig. 1B):

1. Randomly permute the original *P*-value ranks of genes M times. By default, M = 200.
2. Run module enrichment and GO over-representation analyses for the original gene ranks and all permutations.
3. Let $ {A}_i $ be the set of genes in permutation $i$ that:
  - Are in a module of interacting genes that is significantly enriched for low *P*-values.
  - Are annotated with a biological process term enriched among genes in that module.
  Let ${A}_0$ (the candidate FISHNET genes) be the set of genes satisfying these criteria when using the true ranks.
4. When using permutation-based hypothesis test

$j$ = 0.05 × number of genes.

While $j$ ≥ 10, do:

${B}_j$ = the set of top $j$ genes with the smallest *P*-values.

For $i$ = 0 to M:

5)
  $$ {C}_{ij}={A}_i\cap{B}_j $$
  # Compute the expected FDR for genes above rank $j$
6)
  $$ {\mathrm{FDR}}_j=\frac{\mathrm{mean}\Big(\left|{C}_{1j}\right|,\left|{C}_{2j}\right|,,\dots, \left(|{C}_{Mj}|\right)}{\left|{C}_{0,j}\right|} $$

${\mathrm{quantile}}_j$ = quantile of |C_0,*j*_| in |C_1,*j*_|, |C_2,*j*_|, |C_3,*j*_|, …., |C_M,*j*_|.
  If ${\mathrm{FDR}}_j$ <= 0.05 and ${\mathrm{quantile}}_j$ > = 0.99:
  Return FISHNET genes = A*_0_* ∩ B*_j_.*
  Else: $j$ = $j$ – 10.

## Results

We developed the FISHNET algorithm to identify replicable gene-trait relationships missed by standard association analyses. It works by combining gene-level summary statistics with prior biological knowledge encoded in gene–gene interaction networks and gene function annotations. Among the genes with suggestive/significant *P*-values, FISHNET prioritizes genes that cluster in network modules and share common biological functions (see Fig. 1 and Methods). To evaluate FISHNET, we applied it to GWAS gene-level summary statistics from Choobdar *et al*. 2019 [14] (Supplementary Text 1, Supplementary Table S1) and TWAS summary statistics from the LLFS (Supplementary Text 2) and FHS cohorts.

### Finding Significant Hits in Networks performance varies across networks and modularization algorithms

FISHNET was applied to 23 GWAS discovery-replication summary statistics pairs. Each pair was analyzed in combination with nine sets of network modules obtained by applying three modularization algorithms to three networks. Specifically, algorithms based on kernel clustering, modularity optimization, or random walk were applied to the STRING functional PPI network [26], InWeb physical PPI network [27], and a gene co-expression network [28, 29]. Characteristics of the module sets and associated GO terms are provided in Supplementary Table S6.

**Figure 2:**
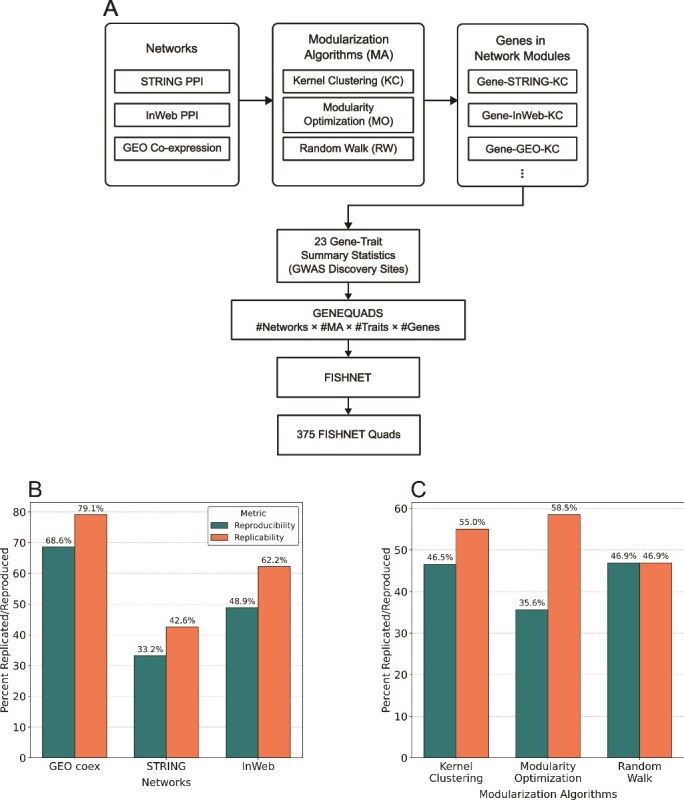
Replication and reproducibility rates across networks and modularization algorithms using 23 pairs of GWAS discovery and replication summary statistics. (A) Three modularization algorithms are applied to three networks to obtain nine sets of gene modules, each fed into FISHNET with gene-trait summary statistics. (B) GEO co-expression outperforms other networks in terms of replicability and reproducibility, while STRING functional PPI performs worst in both metrics. (C) Kernel clustering achieves the best balance of replication and reproducibility, while modularity optimization performs best in replication, and random walk performs best in reproducibility.

Each FISHNET output gene (hit) from each run can be uniquely identified by four factors: the gene, network, modularization algorithm, and trait used in the run. For convenience, we refer to these unique identifiers as *genequads* (Fig. 2A). If a genequad is identified by FISHNET, we refer to such genequad as a FISHNET quad. Each network was evaluated on the reproducibility and replicability of all FISHNET quads based on that network (unioned over the other three factors). Likewise, modularization algorithms were evaluated on all FISHNET quads based on that algorithm, unioned over other factors. A FISHNET quad from a discovery set is reproduced if it is also a FISHNET quad in the replication set; it is replicated if a hit identified in the discovery set is Bonferroni significant in the replication set.

Across 23 GWAS discovery datasets, three networks, and three modularization algorithms, FISHNET identified 375 Genequads, of which 162 (43.2%) reproduced and 200 (53.3%) replicated (Supplementary Table S3). Among networks, the GEO co-expression network had the best reproducibility and replication rate, followed by InWeb PPI (Fig. 2B). Among modularization algorithms, random walk had the best reproducibility rate and modularity optimization had the best replication rate (Fig. 2C). Kernel clustering had the best performance when both replicability and reproducibility were considered. The analyses below are based on the two best-performing networks, InWeb PPI and GEO co-expression, and the best modularization algorithm, kernel clustering.

For each network and modularization algorithm pair, we examined whether network characteristics (number of modules, average module size, number of unique genes in modules) affected the number of FISHNET quads. Across all network and modularization algorithm combinations, none of these characteristics showed a significant association with the number of FISHNET quads (Supplementary Table S6).

### Finding Significant Hits in Networks identifies replicable gene-trait relationships missed by association analyses

We previously published results from association analyses of blood gene expression levels with 11 cardiovascular risk traits in the LLFS cohort [8] . For the current paper, we applied FISHNET to those summary statistics to identify replicable gene-trait relationships that did not reach significance thresholds. Across 11 traits, FISHNET identified 287 unique gene-trait relationships, 34 of which replicated in the FHS cohort (Table 1, Supplementary Table S4). Nineteen of the 34 were not Bonferroni significant in the LLFS cohort (Table 1, Supplementary Table S8). For pulse and Forced Vital Capacity (FVC) there were no significant hits in the original study, but both have one replicated FISHNET gene. For BMI, HDL, and Triglycerides, FISHNET identified 15 replicated gene-trait relationships that were genome-wide significant in the original studies and 17 that were not.

**Table 1:** FISHNET identified replicated gene-trait relationships across five traits in LLFS, including both genes that meet the genome-wide significance threshold and those that do not.

Genes	Trait	Bon. Significant in LLFS (*P* ≤ 3.5 × 10^−6^)
*PTGER2, FFAR2, CX3CR1, HCK, BIRC3, UBE2J1, DUSP2*	BMI	Yes
*TNF, LTB, FLT3, CD22, CLEC4E, MMP8, TLR5, CCR6, SERPINA1, EDA, IL18R1, PEA15*	BMI	No
*MS4A3, SREBF2, GATA2*	HDL	Yes
*CD180, GCA, RUNX1*	HDL	No
	Pulse	Yes
*ITGAM*	Pulse	No
*TCN1, MS4A3, SREBF2, RUNX1,GATA2*	Triglycerides	Yes
*LTB, CD244*	Triglycerides	No
	FVC	Yes
*CD1D*	FVC	No

**Figure 3:**
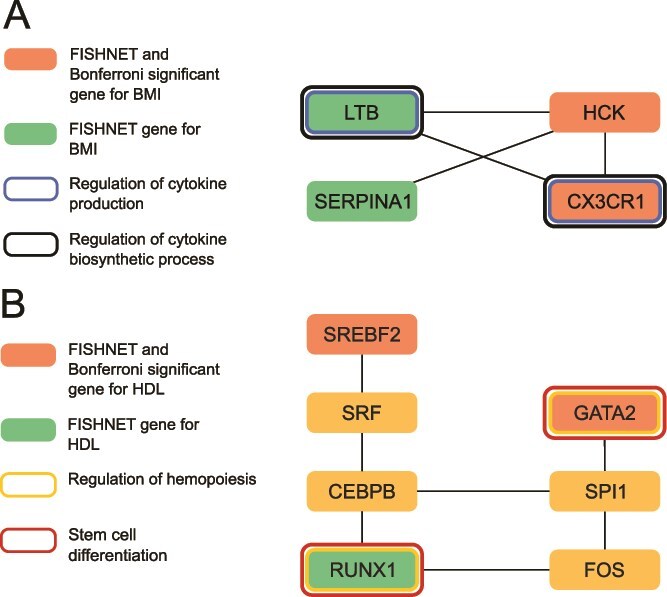
FISHNET genes interact with genome-wide significant genes in significant network modules. (A) A significant co-expression module for BMI contains 4 replicated FISHNET genes. Two of these (*HCK* and *CX3CR1*) are Bonferroni-significant in LLFS and two are not (*LTB* and *SERPINA1*). *LTB* directly interacts with *CX3CR1* and *HCK* and, like *CX3CR1*, participates in cytokine production and cytokine biosynthetic processes. (B) A significant InWeb PPI module for HDL contains a replicated FISHNET gene (*RUNX1*) with 2 Bonferroni-significant and replicated FISHNET genes (*GATA2*, *SREBF2*). *RUNX1* participates with *GATA2* in the regulation of hemopoiesis and stem cell differentiation. *RUNX1* is connected to both *SREBF2* and *GATA2* via two mediator genes.

FISHNET identified an association between expression of *LTB*, the gene encoding lymphotoxin-beta, and BMI, which is strongly supported by experimental studies in mice. *LTB*, a member of the tumor necrosis factor family, regulates immune responses through the activation of the LTβR pathway [30, 31]. Inactivation of this pathway in Ltβr^−/−^ mice confers resistance to diet-induced obesity, possibly through its effects on the gut microbiota [32]. The direction of this effect is consistent with our finding that expression of *LTB* is positively associated with higher BMI in humans. The mouse experiment supports the possibility that higher expression of *LTB* increases BMI by activating the LTβR pathway. Given that cytokine-mediated immune responses are key mechanisms in obesity-induced inflammation [33, 34], obesity may also induce expression of *LTB* in a positive feedback loop. One previous study showed upregulation of *LTB* in peripheral blood monocytes of 14 mildly obese Korean men but not in 12 moderately obese men [35]. However, there is no previous evidence for association between the expression level of *LTB* and BMI in the TWAS Atlas [36], nor is there any previous genetic evidence linking *LTB* to BMI in humans in the GWAS Catalog [37]. Although *LTB* did not reach genome-wide significance for BMI in our TWAS, FISHNET supported an association because: (i) *LTB* is in the same module of the co-expression network with other genes that show evidence of association with BMI, including *SERPINA1*, *HCK*, and *CX3CR1*, and (ii) *LTB* is annotated with GO terms involving cytokine production that are over-represented among genes in that module (Fig. 3A). There is no guarantee that genes with similar expression patterns will be involved in the same molecular processes, but the combined FISHNET criteria highlighted a relationship between *LTB* and *CX3CR1,* both of which are involved in immune signaling. Supporting this, LTB/LTβR signaling has been shown to induce expression of CX3CL1, a pro-inflammatory chemokine ligand for *CX3CR1*, thereby linking LTB activity to *CX3CR1*-mediated pro-inflammatory pathways [38].

FISHNET also identified a novel, positive association between *RUNX1*, the gene encoding the Runt-related transcription factor (TF) 1, and HDL. This finding is strongly supported by experimental evidence highlighting *RUNX1*’s role in promoting white fat browning, which in turn increases HDL cholesterol levels. The RUNX1 protein promotes white fat browning by binding to the promoters of the brown-adipose-tissue-specific genes *Pgc-1α* and *Ucp-1* in inguinal white adipose tissue (iWAT) [39]. Deletion of *RUNX1* (in mice lacking CDK6) reduces the expression level of *Pgc-1α* and *Ucp-1*, consequently inhibiting white-fat browning [39]. Furthermore, inducing white fat browning via Kaempferol (KPF) treatment in mice under both a high-fat diet and normal chow diet enhances RUNX1 protein levels and increases the expression of *Pgc-1α* and *Ucp-1* in iWAT [40]. Chemical induction of white fat browning via KPF treatment has also been directly linked to the change in HDL-cholesterol levels. Specifically, when white fat browning is induced in mice with hypertriglyceridemia (Apoa5−/−), the cholesterol shifts from the triglycerides-rich lipoproteins to HDL, increasing the amount of HDL-cholesterol [41]. The role of *RUNX1* in promoting HDL levels in mice is consistent with our finding that higher expression of *RUNX1* is associated with higher HDL levels in humans.

Module significance analysis links *RUNX1* to cholesterol production via interactions with *SREBF2.* A physical PPI module containing *RUNX1* and *SREBF2* is significantly enriched for genes with low *P*-values for association with HDL (Fig. 3B). *RUNX1* is connected to *SREBF2* via *CEBPB* and *SRF* (Fig. 3B). *SREBF2* upregulates genes involved in cholesterol production, including HMG-CoA reductase, the target of the cholesterol-lowering statin drug family. Statins lower cholesterol by inhibiting HMG-CoA reductase [42, 43]. Interestingly, an intronic variant (rs2834707*)* in *RUNX1* has been previously associated with HDL in the GWAS Catalog [37]. However, there is no prior association of *RUNX1* expression with HDL in the TWAS Atlas [36].

### Finding Significant Hits in Networks genes replicate at a higher rate across *P*-value and false discovery rate thresholds

**Figure 4:**
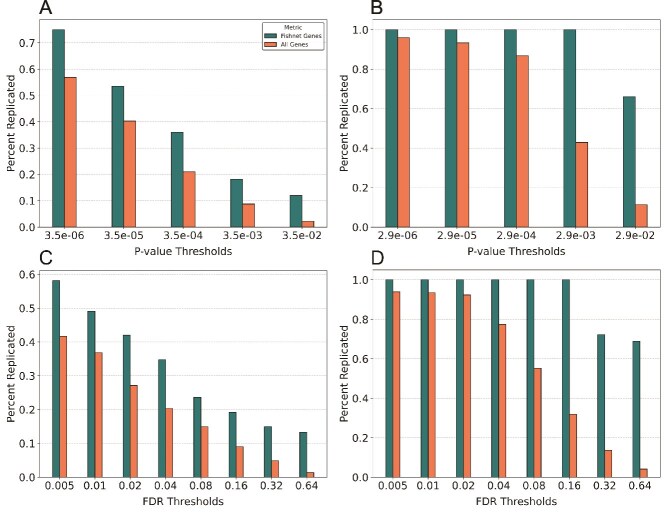
Replication percentage across *P*-value and FDR thresholds. The *x*-axis shows different *P*-values and FDR thresholds. The *y*-axis shows the percentage of genes satisfying the indicated threshold that replicated (A) the replication percentage across *P*-value thresholds in the LLFS cohort (genome-wide significance threshold: p ≤ 3.5 × 10^−6^). (B) The replication percentage across *P*-value thresholds in the GWAS summary datasets (genome-wide significance threshold: p ≤ 2.9 × 10^−6^). (C) and (D) The replication percentage across FDR thresholds in the LLFS cohort and GWAS summary datasets, respectively. Across all *P*-value and FDR thresholds, FISHNET genes have a better replication rate than all genes satisfying the threshold.

We compared the replication rate of all genes satisfying different *P*-value thresholds with the replication rate of FISHNET genes above the same thresholds. In the summary statistics from TWAS of 11 cardiovascular risk traits in the LLFS cohort, the FISHNET genes had a higher replication rate than all genes satisfying the same *P*-value thresholds (Fig. 4A). Specifically, we set a series of *P*-value thresholds, starting with the genome-wide, Bonferroni-corrected significance threshold (p ≤ 3.5 × 10^−6^) and increasing by factors of 10 until we reached p ≤ 3.5 × 10^−2^. Genes were categorized into cumulative bins such that each bin includes all genes satisfying its threshold as well as those satisfying any lower thresholds. The replication rate of FISHNET genes at p ≤ 3.5 × 10^−5^, a factor of ten more liberal than the Bonferroni criterion, was similar to that of all Bonferroni significant genes. More generally, the replication rate of FISHNET genes at each threshold was similar to that of non-FISHNET genes at a one-order-of-magnitude more stringent threshold, a trend observed across all thresholds (Fig. 4A). The same analysis in 23 GWAS discovery datasets showed an even better result (Fig. 4B). Among genome-wide significant genes (p ≤ 3.5 × 10^−6^), those that were also FISHNET genes had a higher replication rate than the rest. We also compared the replication rate of all genes satisfying different FDR thresholds with that of FISHNET genes satisfying the same thresholds (Fig. 4C and D). In the LLFS data, the replication rate of FISHNET genes at each threshold was comparable to that of non-FISHNET genes at an FDR threshold four times more stringent (see the two bars in Fig. 4C). The results from GWAS gene-level summary statistics are even stronger (Fig. 4D). The evidence from both datasets suggests relaxing the FDR threshold by a factor of 4 for FISHNET genes will not compromise the replication rate.

### Finding Significant Hits in Networks is not suitable for co-expression networks built from the same expression data used to generate the *P*-values

**Figure 5:**
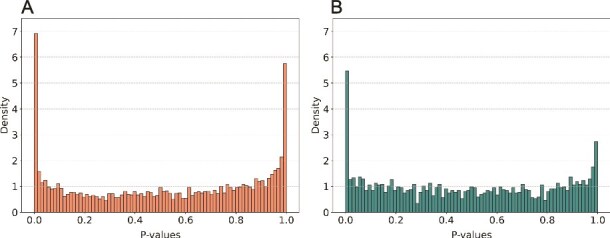
Comparison of module *P*-value distributions across two types of co-expression networks using LLFS TWAS summary statistics as input. (A) The distribution of module *P*-values from the LLFS co-expression network. The distribution has a prominent U-shape in this case. (B) The distribution of module *P*-values from the GEO co-expression network.

The intuition behind FISHNET is that, under the null hypothesis, gene *P*-values should be randomly distributed across the network. We hypothesized that this might not be the case when the gene *P*-values come from association of gene expression levels with traits and the network is generated from the same gene expression data. The reason is that genes in modules of co-expression networks are expected to have similar expression patterns in the data used to generate the network. If the same data are used for association with traits, genes with similar expression patterns across participants can be expected to have similar *P*-values. Therefore, genes in the same module may have similar *P*-values, even when those *P*-values are large and the genes are therefore unlikely to be associated with the trait. To test this hypothesis, we built a co-expression network from the 1810 LLFS gene expression samples by using GENIE3 (https://github.com/vahuynh/GENIE3) [44] (LLFS-net). Modules were identified using the K1 method and submitted to FISHNET, along with *P*-values for association of the same gene expression levels with 11 cardiovascular risk traits. Across the 11 traits, FISHNET identified 162 enriched module-trait pairs, compared to 38 for the co-expression network based on independent GEO data. A histogram of *P*-values across modules showed a U-shaped distribution with substantial over-representation of *P*-values near 1.0 (Fig. 5A), consistent with genes with large *P*-values (bottom ranked) clustering in network modules. The same histogram from the GEO network showed much less over-representation or large *P*-values (Fig. 5B). To further investigate this, we reversed the ranking of genes by *P*-values, so that the most significant genes had the largest *P*-values (bottom ranked) and the least significant ones had the smallest *P*-values (top ranked). Genes that are not truly associated with the trait, which have now been reassigned the smallest *P*-values, should not cluster into modules and so no significant modules should be found. However, when the modules were from LLFS-net, 5 significant modules were found after *P*-value reversal; when they were from the GEO network built from independent data, no significant modules were found. The FISHNET software outputs the number of significant modules after rank reversal as a diagnostic evaluation of the modules’ suitability for the summary statistics. We also developed an empirical statistical test, as an alternative to the number of significant modules upon rank reversal, to quantify the dependency between the summary statistics and network modules. The test is described in Online Supplement and the results of applying it to our data are shown in Supplementary Table S7.

### An alternative thresholding mechanism allows more control over Finding Significant Hits in Networks false discovery rate

**Figure 6:**
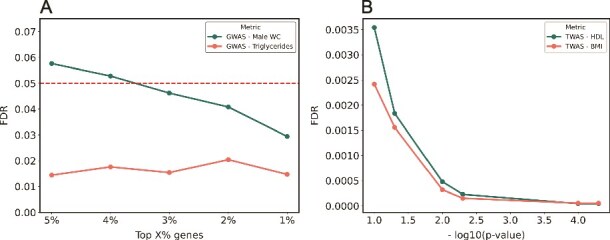
(A) The change in FDR as a function of top X% genes selected in the thresholding mechanism based on iteratively removing genes with the largest *P*-values for GWAS on male waist circumference and triglycerides levels. (B) The change in FDR as a function of module *P*-values in the alternative thresholding mechanism.

When using empirical hypothesis testing, FISHNET only outputs a gene set if it can identify one that meets its thresholds on permutation-based FDR and quantile (Fig. 1). To search for such a set, it first identifies the set ${A}_0$ of all genes that (i) are in a module that is significantly enriched for low *P*-values and (ii) are annotated with a GO biological process term that is enriched among genes in that module. It then intersects ${A}_0$ with ${B}_j$, the set of all genes with the $j$ smallest *P*-values, where the default value of $j$ is 5% of the total number of genes, and tests ${A}_0\bigcap{B}_j$ to see whether it satisfies the FDR and quantile criteria. In all but one of the FISHNET runs reported above in which ${A}_0$ is not empty, ${A}_0\bigcap{B}_j$ does satisfy the criteria. But when it does not, FISHNET tests ${A}_0\bigcap{B}_{j-10},{A}_0\bigcap{B}_{j-20},\dots$ until it finds an intersection that satisfies the criteria. In the run on male waist circumference, this process was necessary and resulted in a satisfactory intersection, ${A}_0\bigcap{B}_{70}$. Figure 6A shows the empirical FDR as a function of the percentage of genes included in $B$ for this trait, and in this case the line is monotonically decreasing as $k$ gets smaller. However, there is no guarantee that it will be—Fig. 6A also shows the FDR line for triglycerides, which bounces up and down with no significant trend. It is therefore possible that the FDR could fail for all values of $j$. To increase the likelihood of identifying a non-empty subset of ${A}_0$ that satisfies the criteria, we implemented an alternative approach.

In the alternative approach, instead of removing genes with the largest *P*-values, we iteratively reduce the threshold on the *P*-value for modules, which is returned by PASCAL (module-based filter, Supplementary Fig. S1). Starting with modules that had Bonferroni-adjusted *P*-values ≤0.1, the candidate FISHNET gene set was iteratively reduced to genes within modules meeting increasingly stringent thresholds until the FDR and percentile criteria were met. Notably, using the module-based filter decreased FDR monotonically as module *P*-value thresholds became more stringent (Fig. 6B). On the LLFS TWAS summary statistics, this alternative method performed comparably to the original FISHNET pipeline in terms of replication rates (Supplementary Fig. S2A and B). On the 23 GWAS summary datasets from Choobdar *et al*. 2019 [14]. FISHNET genes obtained by the alternative method (module-based filter) showed slightly lower replication rates compared to other genes with the smallest gene *P*-values while showing substantially higher replication rates for genes with larger *P*-values (S3A and B).

Overall, iteratively removing genes with large *P*-values outperformed the module-based filter across two datasets and ensured that FISHNET genes met the FDR and percentile rank criteria, establishing it as the preferred method in this work.

## Discussion

Multiple testing correction approaches such as Bonferroni adjustment [1, 2] and FDR control [3, 4] reduce false positives at the cost of increasing false negatives. FISHNET offers a way to relax significance thresholding while maintaining the rate of replication in independent cohorts. Across 11 cardiovascular risk traits in the LLFS cohort, FISHNET identified 34 replicable gene-trait relationships, 19 of which were not genome-wide significant in TWAS according to standard thresholds.

### A robust, multi-purpose tool for gene prioritization

An easy-to-install, easy-to-use implementation of FISHNET is available at https://brentlab.github.io/fishnet/. Given gene-level summary statistics and modules derived from a gene–gene interaction network, it generates (i) a prioritized list of FISHNET genes, (ii) a diagnostic evaluation of the modules’ suitability for the summary statistics, and (iii) a list of significant network modules along with enriched GO terms associated with module genes. FISHNET also outputs expected FDR and the quantile of the number of candidate FISHNET genes identified based on permutation analysis. By default, FISHNET accepts candidate genes with FDR ≤ 0.05 and quantile ≥99%, but users can change this to meet the needs of their application. Users can also customize the pipeline by defining the initial threshold on gene-trait *P*-values required for genes to be considered (default: top 5% of genes, ranked by *P*-values).

To assess FISHNET’s sensitivity to the choice of permutation-based thresholds, we varied the FDR cutoff from 0.01 to 0.10 and the quantile cutoff from 0.80 to 0.99 (see Online Supplement for details). Across this entire range of cutoffs, the total number of FISHNET genes barely changed (Supplementary Table S5), confirming the robustness of FISHNET. This is expected because FISHNET internally uses PASCAL to identify modules that contain a statistically significant clustering of genes with low *P*-values from gene-trait associations. It selects modules only if their module *P*-values, which PASCAL calculates using a chi-squared test, are less than 0.05 after Bonferroni correction for the number of modules in the network. Because this threshold is stringent, PASCAL rarely returns modules in which the clustering of low gene-level *P*-values occurs by chance. Thus, random permutation runs tend to return no significant modules, so the default thresholds on the permutation-based significance criteria are usually satisfied. The number of FISHNET genes is typically reduced only when users set thresholds that are much more stringent than the defaults, such as FDR < 0.01. The number of FISHNET genes also remained stable across different numbers of random permutations from 200 to 5000, indicating that the default setting of 200 permutations in FISHNET software is enough to get stable results.

### Relationship to other methods for post-processing genome-wide association studies results

The goal of FISHNET is to identify a small set of exceptional, trait-influencing genes and to recommend them for replication studies or other follow-ups, even when their *P*-values from gene-trait association are not significant by conventional criteria. One step in the FISHNET process is to identify network modules that are enriched for genes with low *P*-values. FISHNET uses a method called PASCAL to do this, but there are many possible alternatives, including gene set enrichment analysis [45], over representation analysis, and other methods reviewed in ref. [46]). Some of these methods could be substituted for PASCAL, but they are not comparable to FISHNET because they do not recommend individual genes for follow-up.

FISHNET makes use of *P*-values from gene-trait association together with external information about genes—gene–gene networks and curated gene function annotations. Many other methods aim to increase the power of GWAS by incorporating external information about individual SNPs, including SKAT [47], STAAR [48], and other methods reviewed in ref. [49]. Because these methods focus on individual SNPs, they cannot use the gene-level information that FISHNET uses. They also cannot be applied in situations where there are gene-level *P*-values but no SNP-level *P*-values, such as TWAS with measured gene expression levels or differential expression analysis. Two other methods, stratified FDR [50] and functional FDR [51], can be applied to individual SNPs or genes. However, the external information stratified FDR uses consists of non-overlapping gene categories, usually defined by ranges of a numerical variable. Functional FDR uses a numerical variable directly. Neither method can use the gene–gene networks or biological function annotations that FISHNET uses.

FISHNET also improves on traditional network-based methods for association analysis in both methodology and evaluation. One of the most popular approaches is to select previously known trait-associated genes as seed nodes and propagate their scores across local neighborhoods in the network to predict new gene-trait relationships [52, 53]. This approach creates a bias against detecting genes that affect the trait via pathways that are different from those of known genes. FISHNET uses all gene-level *P*-values, which inherently reduces pathway bias, and it identifies modules that are significantly enriched for genes with low *P*-values, even if those genes are far from significant when considered individually. Furthermore, FISHNET incorporates GO biological process annotations to identify genes that (i) interact in significant network modules and (ii) participate in a common biological process. In terms of evaluation, the most common evaluation metrics to validate network-based approaches are leave-one-out methodology [53, 54] and validation with genes from published drug target databases [55]. These metrics test the methods’ ability to perform well in recovering *known* gene-trait relationships. However, the ultimate goal is to uncover *novel* gene-trait relationships. To achieve this, we evaluated FISHNET by comparing the replication rate of FISHNET genes against those meeting standard Bonferroni or FDR thresholds.

### Findings from Finding Significant Hits in Networks and recommendations

Our results suggest best practices for using FISHNET. Among the modularization algorithms tested, we recommend the kernel clustering method K1 [14] when prioritizing both reproducibility and replicability. The modularity optimization method, M2, performed best in replicability, while the random walk approach performed best in reproducibility (Fig. 2B). As an alternative, one could use several modularization algorithms. The analyst’s goal determines how to combine the results: taking the union of the identified genes will yield a broader set, whereas taking the intersection will produce a more conservative, core set of high-confidence genes. While we did not evaluate the replicability and reproducibility of FISHNET genes as a function of the number of modularization algorithms used, all three algorithms we tested performed well in at least one evaluation metric. This suggests that combining results from multiple algorithms is a promising strategy.

Among networks, FISHNET performed well on the InWeb physical PPI and GEO co-expression networks, but poorly on the STRING functional PPI network (Fig. 2A). We recommend using networks based on physical PPI and co-expression interactions over those based on other functional interactions. We also caution against using the same dataset to generate summary statistics and construct gene-based networks, as this can lead to unreliable FISHNET gene-trait relationships (Fig. 5A and B). Based on the results shown in Fig. 4, we recommend using a slightly relaxed Bonferroni or FDR-based threshold and prioritizing FISHNET genes that meet these relaxed criteria alongside the genes that are significant under the original threshold. This recovers gene-trait relationships missed by standard thresholds while maintaining the replication rate.

### Limitations and opportunities for broader applications

There are many potential use cases for FISHNET that have not yet been validated. First, the gene-level *P*-values can come from any source, including RNA-Seq experiments (such as comparing cells treated with a drug to untreated cells) and CRISPR screens to identify genes that affect cellular traits [56, 57]. Second, FISHNET has been tested only on specific gene–gene networks, but others might give better, worse, or complementary results. For example, FISHNET was tested on co-expression networks, but these are neutral as to the molecular mechanism that causes each pair of linked genes to have similar expression patterns. A mechanistic alternative is networks that link TFs to their direct targets [58–61]. Modules from mechanistic gene regulatory networks could elucidate the specific TFs mediating gene–trait relationships. Third, FISHNET has only been tested on modules identified by three algorithms. Since different traits and networks performed optimally with modules from different algorithms, it might be valuable to test a broader range of modularization strategies. More generally, FISHNET uses only the set of genes in each module, not its internal connectivity. Thus, biologically coherent gene sets from any source could be used. For example, the sets of genes directly regulated by each TF could be used, in which case no modularization algorithm is required. Fourth, the final stage of FISHNET uses GO Biological Process terms to identify biological functions enriched among genes in significant modules, but gene sets from other sources could be used, too. For example, gene sets defined by drug target discovery databases such as DrugBank [62, 63], DisGeNET [64], and Open Targets [65, 66] could provide complementary insights and reveal new genes. The versatility of our implementation allows users to try out any of these sources for gene-level *P*-values, module gene sets, and functional gene sets. Future work validating FISHNET with new knowledge sources will greatly expand its applicability.

**Key Points**

- Finding Significant Hits in Networks (FISHNET) integrates gene-level summary statistics with prior biological knowledge from network modules and functional annotations, and is specifically designed to identify replicable gene-trait associations that may be missed by traditional genome-wide significance thresholds.
- FISHNET identified gene-trait associations that replicate at higher rates than other associations with similar *P*-values, enabling relaxation of significance thresholds without reducing replication rate.
- Applied to the Long Life Family Study cohort, FISHNET identified 17 replicated gene-trait associations across 11 cardiovascular risk traits that were missed by transcriptome-wide association study and Bonferroni-corrected thresholds.
- FISHNET software is freely available at https://brentlab.github.io/fishnet/.

## Supplementary Material

Supplementary_materials_bbag061

## Data Availability

The FISHNET pipeline is available at https://brentlab.github.io/fishnet/. The gene-level summary statistics from association analyses based on the LLFS cohort and the 2019 Disease Module Identification DREAM Challenge, along with the corresponding FISHNET outputs, are available in the Supplementary Files. The datasets used to generate the LLFS summary statistics and the inputs and outputs from FHS association analyses have not been deposited in public repositories due to data use constraints.
