# Supplementary material for: Finding Significant Hits in Networks: a network-based tool for analyzing gene-level P-values to identify significant genes missed by standard methods
Source: Brief Bioinform. 2026 Mar 8;27(2):bbag061. doi: 10.1093/bib/bbag061 (PMC12967332; doi:10.1093/bib/bbag061)
Supplement: Supplementary_materials_bbag061 [file supplementary_materials_bbag061.zip › Supplementary_Text_and_Figures.docx]

**Supplementary Text:**

1. **Sensitivity Analysis of FDR, Percentile‑Rank Thresholds, and Number of Random Permutations**

To determine how sensitive the FISHNET outputs are to the FDR and PercentileRank Thresholds, we varied them and reran FISHNET on 34 trait datasets (23 GWAS, 11 LLFS TWAS) using three networks for each (102 total FISHNET runs). We varied the percentile-rank threshold from 80% to 99% with a fixed FDR of 0.05 and observed no change in the FISHNET gene set. When we varied the FDR threshold from 0.1 to 0.01 at a fixed percentile-rank of 99%, only the most stringent threshold, 0.01, changed the set of FISHNET genes identified, reducing the sum of FISHNET genes returned by all 102 runs from 429 to 399. Thus, the results are not very sensitive to these parameters. Intuitively, the reason is that the process of generating candidate FISHNET genes is already rigorous, using a Bonferroni corrected P-value for identifying modules that contain a significant clustering of genes with small P-values. Because the candidate generation process is so rigorous, the candidate sets produced tend to pass our permutation-based tests over a wide range of thresholds. FISHNET uses random permutations as an extra layer of protection against rare cases in which the assumptions used during candidate generation may not hold and to provide the user with the ability to set even more stringent thresholds than are used during candidate generation, such as FDR=0.01.

The number of random permutations of gene labels used to generate a distribution of candidate FISHNET genes under the null hypothesis (used for empirical hypothesis testing) was also varied from 200 to 5000 with an FDR ≤ 0.05 and a percentile rank ≥ 99%. The FISHNET gene sets remained unchanged across different numbers of permutations. Therefore, 200 permutations are enough to get stable results and the 5000 we used in our experiments are more than sufficient.

1. **Empirical Hypothesis Testing to Quantify compatibility of summary statistics and network**

The null hypothesis that highly ranked genes after rank reversal (that is, those with the highest p-values) do not cluster within modules was tested using two datasets. First, LLFS TWAS summary statistics for each of the 11 traits were used as input with LLFS-based co-expression modules. Second, the same summary statistics were used with the GEO co-expression modules. The test statistic was defined as the sum of the negative logarithms of the Bonferroni-corrected p-values for all modules:

$$\sum_{i} -log(\min\left( 1, P_{i}*numModules \right))$$

where the sum is over all modules, $P_{i}$ is the p-value of the $i^{\mathrm{th}}$ module, as calculated by PASCAL, and $\mathrm{numModules}$ is the number of modules in the network. This counts only the significant and near-significant modules because the $i^{\mathrm{th}}$ module contributes zero to the sum if its p-value is greater than 1/ $\mathrm{numModules}$. Next, five thousand random permutations of the gene p-values were generated, and the test statistic was computed for each permutation. The empirical p-value was calculated as the fraction of permutations whose statistic exceeded the value obtained from the actual reversed ranks. When LLFS-based co-expression network modules were used, the analysis of 11 LLFS traits showed that empirical P-value ≤ 0.01 resulted in one significant module after rank reversal, while empirical P-value > 0.02 resulted in none (Supplementary Table S7). Therefore, a threshold of P-value ≤ 0.02 is recommended to detect bias between summary statistics and network modules. The P-value threshold is user-defined and can be made more or less stringent as needed. Applying the same approach to the GEO co-expression network, no trait had an empirical P-value ≤ 0.02 or any significant modules (Supplementary Table S7), indicating that GEO modules are suitable inputs for FISHNET.

**Supplementary Figure Captions:**

**Supplementary Fig 1:** The gene-level omics-WAS summary is fed into module enrichment analysis, which is performed by PASCAL. PASCAL outputs significant modules and their p-values. Gene ontology over-representation analysis identifies biological processes with significant over-representation among genes in each significant module. (B) The workflow illustrates the gene prioritization mechanism for identifying FISHNET genes by applying the thresholding based on module p-values. When the null hypothesis is rejected, a more stringent module p-value is picked iteratively, and filters are re-applied to identify candidate FISHNET genes for empirical hypothesis testing.

**Alt text:** A workflow diagram in two panels. Panel A shows the process of feeding gene-level omics-WAS data into PASCAL for module enrichment, resulting in significant modules and Gene Ontology results. Panel B illustrates the iterative gene prioritization process, showing the thresholding based on module p-values to identify candidates for hypothesis testing.

**Supplementary Fig 2:** Replication rate across P-value and FDR thresholds using the thresholding based on module p-values. The X-axis shows different p-values and FDR thresholds. The y-axis shows the percentage of replicated genes within the corresponding replication set at a given threshold. (A) shows the replication rate across p-value thresholds in the LLFS cohort (genome-wide significant threshold: p ≤ 3.5 × 10^-6^). (B) shows the replication rate across FDR thresholds in the LLFS cohort).

**Alt text:** Two line graphs for the LLFS cohort datasets demonstrating results derived from the module p-value thresholding mechanism. Panel A plots the gene replication percentage against p-value thresholds, and Panel B plots the replication percentage against FDR thresholds.

**Supplementary Fig 3:** Replication rate across P-value and FDR thresholds using the thresholding based on module p-values. The X-axis shows different p-values and FDR thresholds. The y-axis shows the percentage of replicated genes within the corresponding replication set at a given threshold. (A) shows the replication rate across p-value thresholds in the GWAS summary datasets (genome-wide significant threshold: p ≤ 2.9 × 10^-6^). (B) shows the replication rate across FDR thresholds in the GWAS summary datasets.

**Alt text:** Two line graphs for the GWAS summary datasets demonstrating results derived from the module p-value thresholding mechanism. Panel A plots the gene replication percentage against p-value thresholds, and Panel B plots the replication percentage against FDR thresholds.
